# Adenoid Cystic Carcinoma of the Breast: Two Case Reports

**DOI:** 10.7759/cureus.42757

**Published:** 2023-07-31

**Authors:** Chancée L Forestier, Asilis J Defran, Nafisa Kuwajerwala

**Affiliations:** 1 Obstetrics and Gynecology, Ascension Providence Hospital, Southfield, USA; 2 General Surgery, Ascension Providence Hospital, Southfield, USA; 3 Breast Surgery, Ascension Providence Hospital, Southfield, USA

**Keywords:** breast cancer prognosis, surgical interventions, breast mass, rare breast cancer, adenoid cystic carcinoma

## Abstract

In this series of case reports, we present two women diagnosed with rare cancer, adenoid cystic carcinoma (ACC) of the breast. Though this neoplasm has a relatively favorable prognosis, more information regarding its pathophysiology, response to variable treatments, and long-term prognosis is needed. The objective of our case series is to present the medical decisions and clinical courses of a 50-year-old female and a 79-year-old female with ACC of the breast. Through ongoing shared knowledge from cases like ours and further experimental research, a more reliable diagnostic and treatment algorithm can be substantiated.

## Introduction

Adenoid cystic carcinoma (ACC) is a malignant glandular-based neoplasm that is most commonly found in the salivary glands. It is rarely found in the breast, with less than 0.1% of all breast cancers categorized as ACC [1]. In these ACC of the breast cases, the lesion is most often located peri-areolar or in the upper outer quadrant of the breast [2-4]. The most at-risk patient demographic is Caucasian women in their sixth decade of life [2].

This cancer can be distinguished into three subgroups based on its growth pattern: tubular, cribriform, or solid. Although the subgroups are distinct, they can all exist in the same mass, and almost all cases of ACC have a MYB-NFIB or MYBL1-NFIB gene fusion component [5]. The lesions with solid components tend to be more locally aggressive, with the percentage of solid areas sometimes used for grading purposes and prognostic estimations [6]. In regard to breast cancer classification, ACC masses of the breast tend to be triple negative: estrogen receptor (ER) negative, progesterone receptor (PR) negative, and human epidermal growth factor receptor 2 (HER2) negative [7]. However, compared to most other triple-negative breast cancers, ACC is not aggressive, with low morbidity and mortality rates [1]. 

ACC does not have characteristic patterns on imaging, prompting a long differential list until a biopsy is performed [2-7]. Due in part to its rarity, there is much to be learned regarding its carcinogenesis and progression, along with how best to treat this cancer. There is no set diagnostic or treatment algorithm, though surgery is currently the most common treatment pursued [7,8]. In an effort to increase knowledge of this rare breast cancer, we present a case series regarding two patients with ACC of the breast. 

## Case presentation

Case 1 report 

A 50-year-old female, gravida 2 para 2 (G2P2), with a medical history of hypothyroidism, presented to her clinic with concerns about a right upper breast lump. The patient had a past surgical history of endometrial ablation, partial thyroidectomy, and tonsillectomy. Her family history was noncontributory, including negative for breast cancer. On physical exam, she had ecchymosis of the right breast and dense breast tissue bilaterally with prominent fibroglandular foci; however, no discrete or suspicious mass was detected. There were no palpable cervical, supraclavicular, infraclavicular, or axillary adenopathy bilaterally.

**Figure 1:**
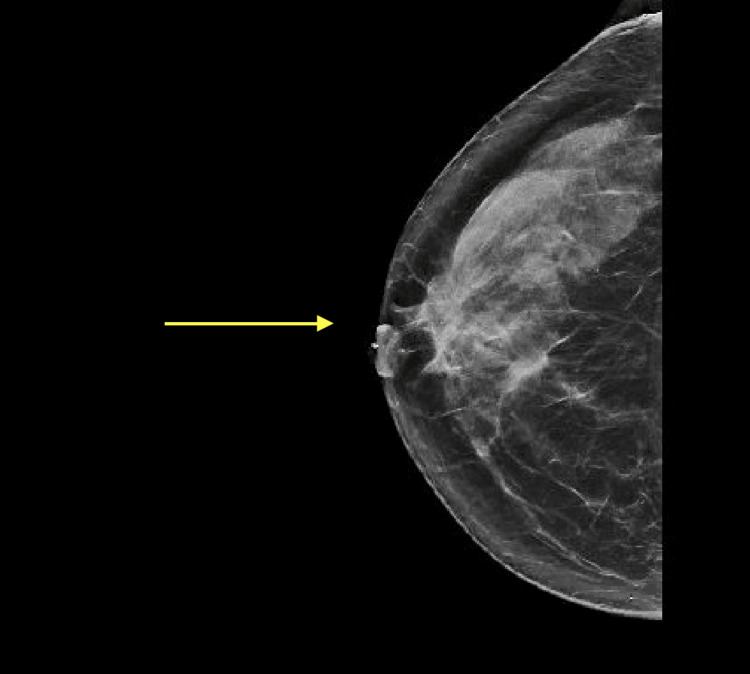
Right-sided diagnostic mammogram Right-sided diagnostic mammogram stated that "There is a possible mass in the right breast at six o'clock anterior depth. There also is possible architectural distortion in the right breast at 10 o'clock anterior depth. No other significant masses, calcifications, or other findings are seen in either breast."

**Figure 2:**
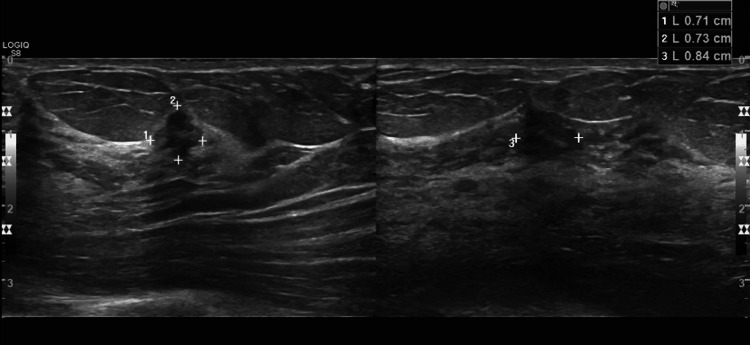
Ultrasound of the 10 o'clock mass Ultrasound findings stated that "The 0.7 cm x 0.7 cm x 0.8 cm mass in the right breast at 10 o'clock middle depth appears suspicious of malignancy."

**Figure 3:**
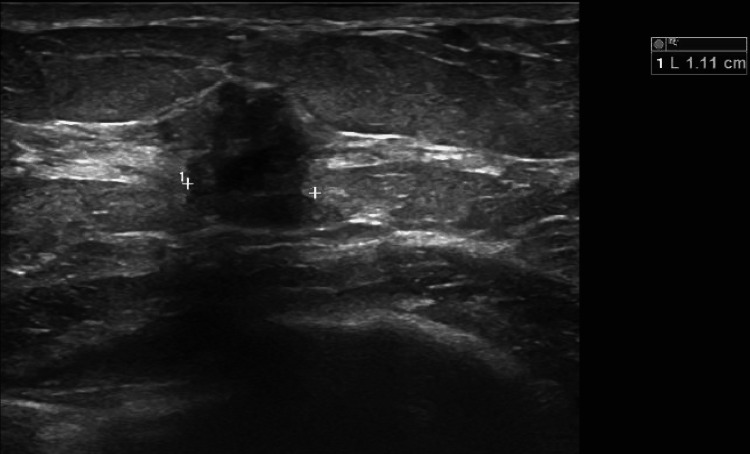
Ultrasound of the six o'clock mass Ultrasound findings stated that "The 1.5 cm x 1.2 cm x 1.1 cm mass in the right breast at six o'clock anterior depth appears suspicious of malignancy."

Due to the patient's history of extremely dense breasts bilaterally on screening mammography, which can disguise pathology, a bilateral diagnostic mammogram was prompted. The diagnostic mammogram did not show an abnormality corresponding to the patient-reported lump; however, imaging was positive for a possible mass in the right breast at six o’clock and architectural distortion at ten o’clock, shown in Figure 1. The mammogram concluded that the breasts were heterogeneously dense and would benefit from an ultrasound. Follow-up ultrasound on the areas of interest demonstrated a 0.7 cm x 0.7 cm x 0.8 cm mass in the right breast at the ten o’clock position, middle depth, 4 cm from the nipple (shown in Figure 2), and a 1.5 cm x 1.2 cm x 1.1 cm mass in the right breast at six o’clock anterior depth (shown in Figure 3). No abnormalities were seen sonographically in the right axilla.

**Figure 4:**
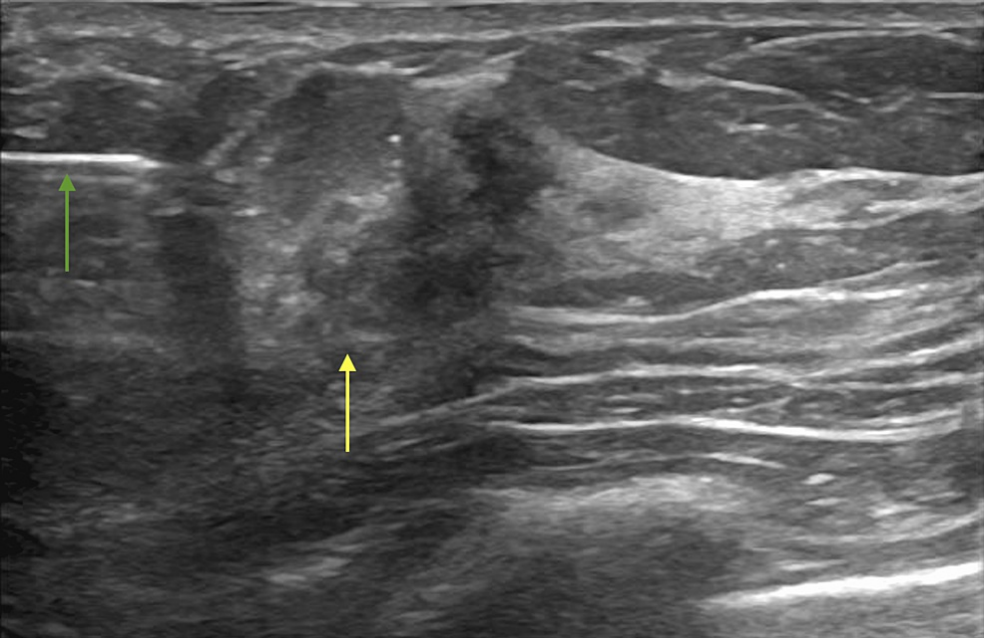
Ultrasound-guided biopsy of the 10 o'clock mass Green arrow: biopsy needle Yellow arrow: mass

**Figure 5:**
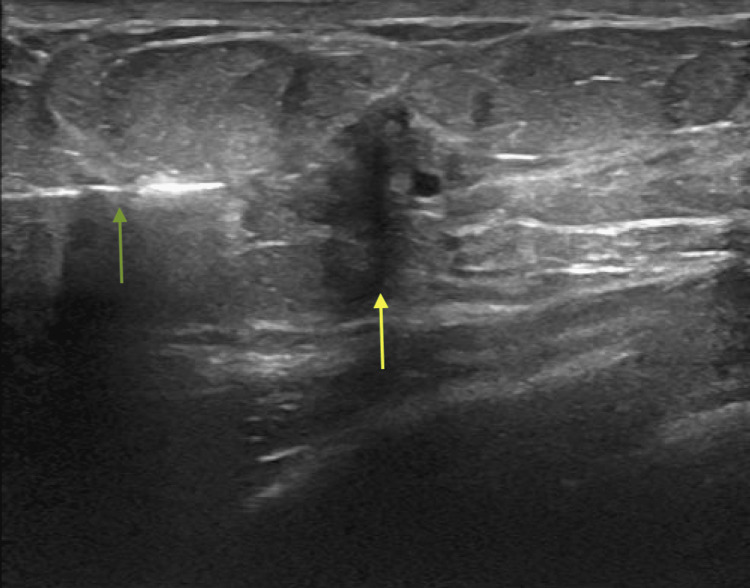
Ultrasound-guided biopsy of the six o'clock mass Green arrow: biopsy needle Yellow arrow: mass

**Figure 6:**
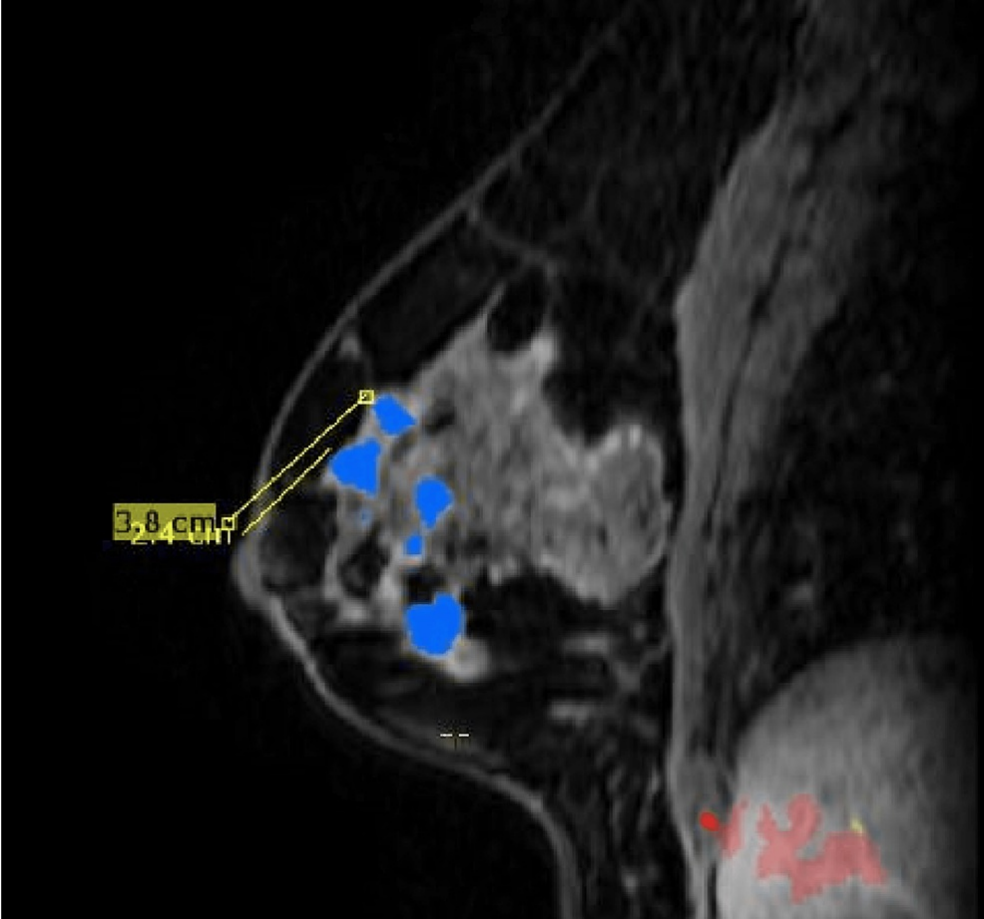
Additional masses found at one o'clock and 12 o'clock MRI findings stated that "At the one o'clock position, approximately 2.4 cm from the nipple, there is a rounded mass, measuring 0.8 cm. At the 12 o'clock position, approximately 3.8 cm from the nipple, there is a rounded mass, measuring 1 cm."

**Figure 7:**
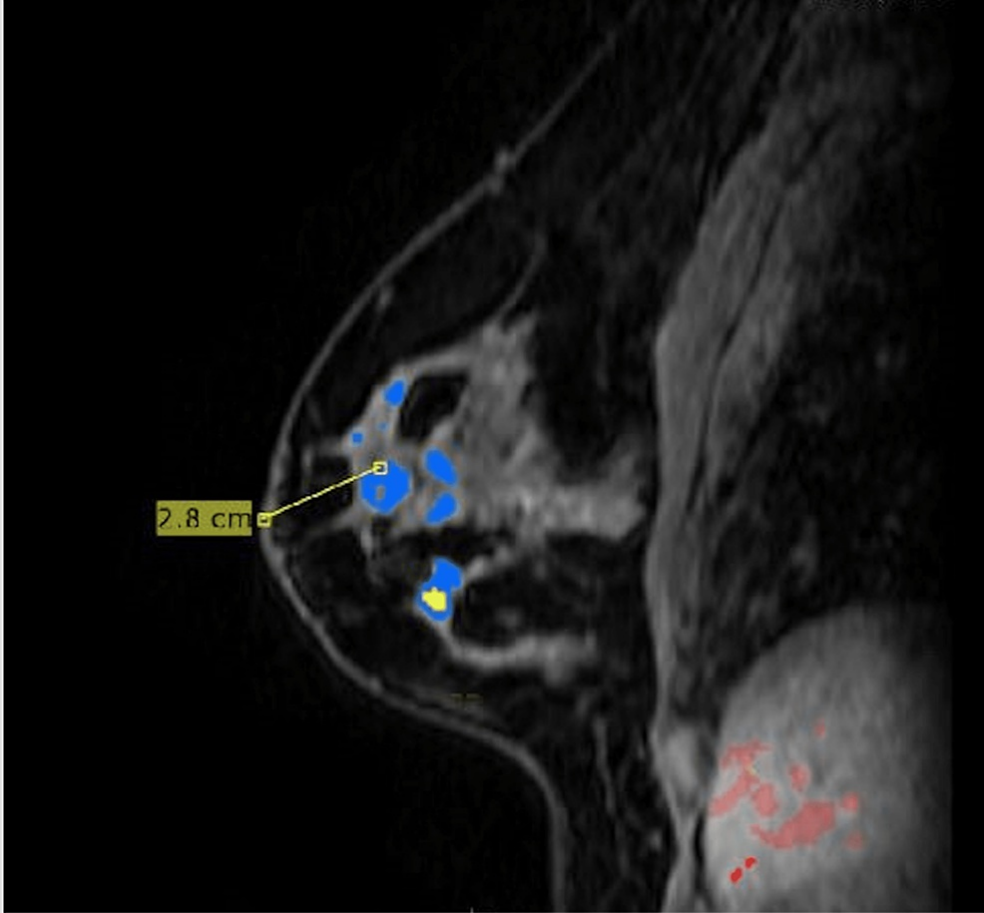
Additional mass found at 11 o'clock MRI findings stated that "At the 11 o'clock position, approximately 2.8 cm from the nipple, there are two adjacent masses, measuring 2.0 in diameter."

The patient then went for an ultrasound-guided biopsy. The biopsy of the 10 o’clock mass showed benign fibroglandular tissue (shown in Figure 4), whereas the six o’clock mass showed invasive ductal carcinoma, grade 1 (shown in Figure 5). Testing was ER positive, PR positive, and HER2/neu negative. Magnetic resonance imaging (MRI) for pretreatment evaluation revealed biopsy-proven carcinoma of the right breast. Additional masses were found, implying multicentric disease. These masses are shown in Figures 6 and 7.

**Figure 8:**
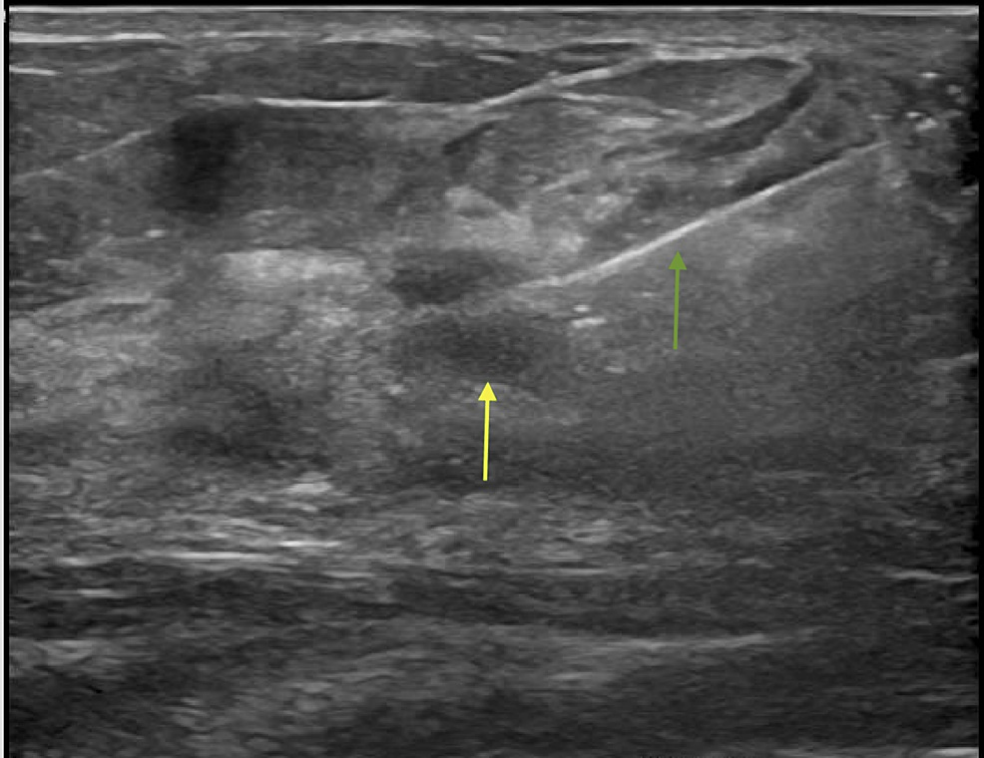
Ultrasound-guided biopsy of the 12 o'clock mass Ultrasound findings stated that "biopsy of the 1.2 cm mass in the right breast at 12 o'clock anterior depth was successful. Pathology indicates adenoid cystic carcinoma. A mass demonstrated at 11 and one o'clock anteriorly on MRI could not be found by US." Green arrow: Biopsy needle Yellow arrow: Mass

When the patient returned for an ultrasound-guided biopsy of the 11, 12, and one o’clock masses, only the 12 o’clock mass could be successfully located and biopsied, as shown in Figure 8. Biopsy results of the 12 o’clock mass returned as ACC, ER/PR negative. At this juncture, an MRI-guided biopsy was recommended if breast conservation surgery was being considered.

After an extensive conversation regarding her therapeutic options, the patient decided to undergo a nipple-sparing right mastectomy with sentinel lymph node biopsy and mammoplasty reconstruction. The final pathology report confirmed multicentric invasive carcinomas, specifically with ACC (ER negative, PR negative, HER2/neu negative), along with invasive carcinoma. The two lymph nodes biopsied were negative for metastatic carcinoma.

Intraoperative angiography monitoring showed poor perfusion to the lower hemisphere of the breast from the nipple inferiorly to the inframammary incision. Ultimately, the decision was made to abort any implant-based reconstruction and perform this in a delayed fashion in two weeks. The patient was discharged postoperative day one with a Jackson-Pratt drain and instructed to follow up for reconstruction. In total, the time elapsed from diagnostic mammogram to surgical resection was 60 days. The patient returned three weeks later for re-excision of the 12 o’clock ACC due to a positive retro-areolar margin, along with the placement of a tissue expander. She continued to follow up with her long-term treatment teams.

Case 2 report 

A 79-year-old female, G3P2, presented to the clinic due to a palpable left breast mass. She had a past medical history of seizures and hypertension. Twenty years prior, she had a left breast biopsy due to a lump, which was shown to be benign. There was a family history of unknown cancer in her deceased father and pancreatic cancer in her brother. On physical exam, her breasts appeared symmetric, while the patient was in a sitting position. However, when the patient was in the supine position, a 2.5-3 cm superficial lump was palpable in the left breast at the 12 o’clock position, 10 cm from the nipple. The mass was mobile and nontender. The right breast examination was benign. There were no palpable cervical, supraclavicular, or axillary lymphadenopathy bilaterally. 

**Figure 9:**
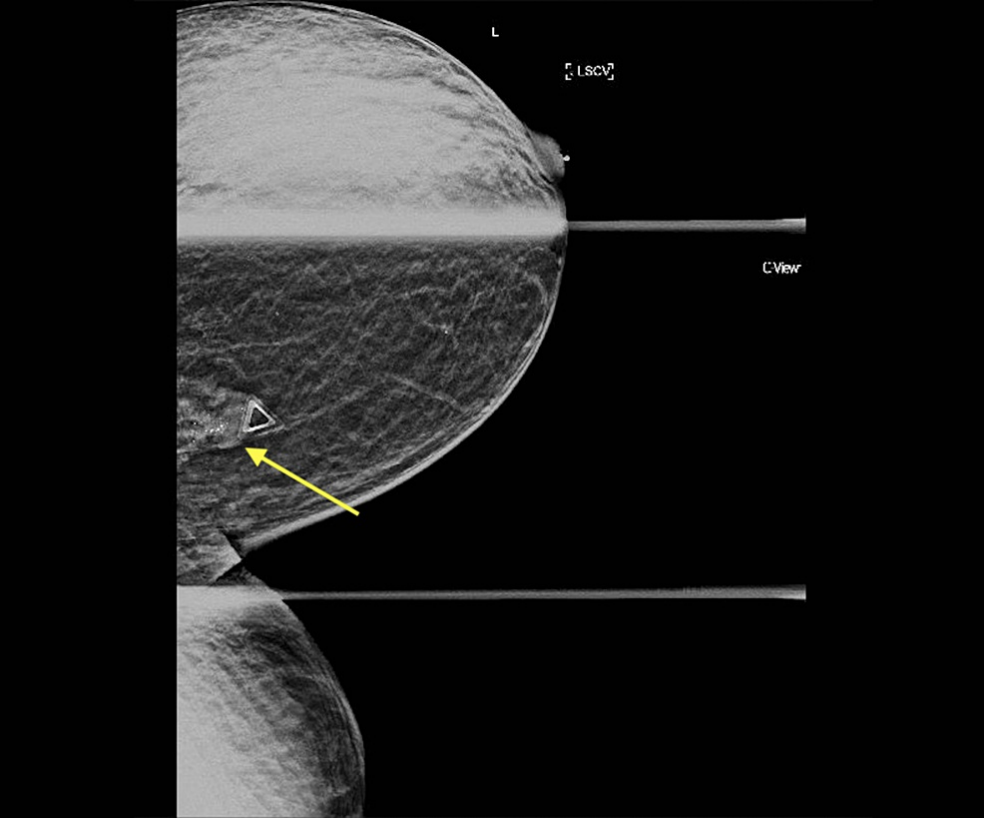
Diagnostic mammogram Ultrasound findings stated that "There is a mass with calcifications in the left breast upper inner aspect posterior depth. This correlates as palpated. No other significant masses, calcifications, or other findings are seen in either breast." Yellow arrow: mass with calcifications

**Figure 10:**
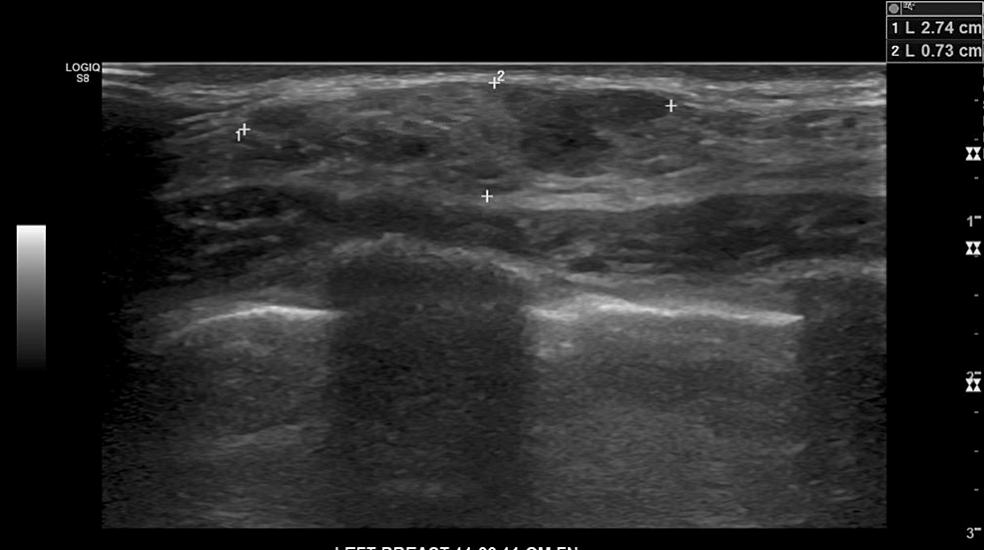
Ultrasound Ultrasound findings stated that "The 27 mm x 16 mm x 10 mm lobulated mass in the left breast at 11 o'clock appears suspicious of malignancy."

**Figure 11:**
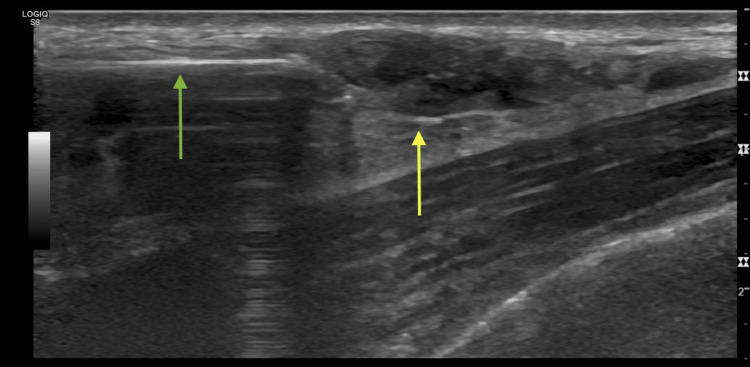
Ultrasound biopsy Ultrasound biopsy findings stated that "The mass in the left breast at 11 o'clock, 11 cm from the nipple, was successful. Pathology indicates malignant invasive adenoid cystic carcinoma (ADC). Pathology results are concordant with imaging findings." Green arrow: biopsy needle Yellow arrow: mass

**Figure 12:**
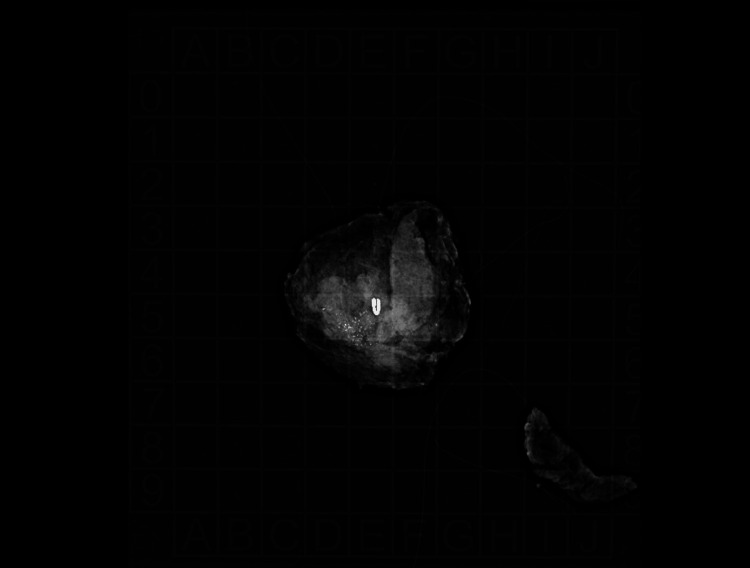
Surgically excised specimen The specimen findings stated that "The imaged specimen includes the mass, calcification, and a biopsy clip."

Diagnostic bilateral mammography demonstrated a mass with calcifications in the left breast, in the upper inner aspect with a posterior depth, as shown in Figure 9. This mass correlated with the palpated mass. The patient then had an ultrasound performed, showing a 27 mm x 16 mm x 10 mm lobulated mass in the left breast at 11 o’clock, 11 cm from the nipple, as shown in Figure 10. The mass was of mixed echogenicity and correlated with the palpated mass and mammography results. It also showed increased vascularity, but no ipsilateral axilla abnormalities were discovered. This was followed by an ultrasound-guided core needle biopsy, resulting in invasive carcinoma, suspected to be solid-type ACC of the breast, as shown in Figure 11. The mass was ER negative, PR negative, and HER2 positive. The patient and provider decided left breast lumpectomy, with sentinel lymph node biopsy, as the patient’s best plan of action. Two sentinel lymph nodes were excised from the left axilla. These were negative for metastatic carcinoma. The lumpectomy demonstrated ACC, solid type with patchy comedonecrosis and calcification of 27 mm x 26 mm x 8 mm in size. The margins included the following: anterior <0.5 mm, posterior <0.5 mm, inferior-posterior <0.1 mm, superior <0.5 mm, and lateral 7 mm. Lastly, the mass was ER, PR, and HER2 negative. Since it had tested positive for HER2 previously, the surgeon ordered HercepTest, and the mass was confirmed to be HER2 negative. She was sent home with instructions to follow up. The excised mass is shown in Figure 12.

Following up with the patient four weeks after the procedure, the patient stated she felt she was healing well, and her energy was at baseline. She denied any pain, shortness of breath, cough, or any new or concerning symptoms since the surgery. Incision sites were well healed without any induration or erythema. Due to the numerous close margins on pathology, computed tomography (CT) simulation with adjuvant radiotherapy was recommended to the patient, along with the risks and benefits. The patient wanted time to think about her decision. Two weeks later, she returned with the wish to begin radiation. Adjuvant local radiation was performed daily Monday through Friday, for four weeks. The patient completed the therapy and experienced some fatigue and local irritation and continued to follow up with her long-term treatment teams. 

## Discussion

ACC is a rare cancer subtype of the breast that most often presents as a palpable mass without accompanying pain. This held true with our two breast cases of ACC, in which both women presented with concerns of non-painful palpable lumps within the breast. ACC of the breast is most often found in adult Caucasian women, ranging from the third to eighth decades of life, with a mean age of diagnosis of 63 years [2,3]. This glandular carcinoma is most commonly found in salivary glands but can also be found in the breast, lung, skin, cervix, larynx, and Bartholin glands [1]. The treatment modalities implemented in these ACC cases with varied locations focus on surgery as the cornerstone with adjuvant radiation therapy depending on staging and histologic irregularity [9]. Within the breast, the ACC masses are most often 1-3 cm in diameter and found in peri-areolar regions, especially in the upper-outer quadrant [2-4]. With metastasis and recurrence being rare, ACC of the breast has a good prognosis. When it does metastasize, it can infiltrate the axillary lymph nodes locally and lungs distally, though other destinations are also possible [1]. Recurrence is more likely when the patient did not undergo radiation [1]. 

Imaging of the mass has shown variabilities and therefore does not have a characteristic pattern: mammographically, it has been described as smooth, irregular, and asymmetric; ultrasonographically, it has been described as a heterogenous or hypoechoic irregular mass; on magnetic resonance imaging, the masses have been described as lobular, irregular, and with or without spiculated margins [2-7]. Most often, patients with ACC of the breast undergo mastectomy with no axillary dissection [2]. Unlike other triple-negative breast cancers, ACC does not tend to be aggressive. Recurrence rates of ACC of the breast after local excision range from 6-37%, though margin status was often omitted from the case reports [10]. Additionally, radiation has been shown to be a positive predictor of survival [8]. Some studies show no recurrence when radiation was used after surgery [11]. Therefore, breast-conserving surgeries followed by radiation may have a bigger role in therapy than currently performed.

## Conclusions

In conclusion, ACC is a glandular type of locally aggressive cancer that in rare cases can affect the breast. The medical community is continuing to learn about these subtypes of occurrences, but as of now, this diagnosis carries a favorable prognosis. Although this breast cancer has low morbidity and mortality rates, more information is needed in order to provide a universal treatment plan. Once more knowledge regarding therapy options and long-term outcomes is observed in more patients, physicians and surgeons will be able to propose the best and most reliable treatment plan for their patients.

## References

[1] Glazebrook KN, Reynolds C, Smith RL, Gimenez EI, Boughey JC (2010). Adenoid cystic carcinoma of the breast. AJR Am J Roentgenol.

[2] Pia-Foschini M, Reis-Filho JS, Eusebi V, Lakhani SR (2003). Salivary gland-like tumours of the breast: surgical and molecular pathology. J Clin Pathol.

[3] Thomas DN, Asarian A, Xiao P (2019). Adenoid cystic carcinoma of the breast. J Surg Case Rep.

[4] Kashiwagi S, Asano Y, Ishihara S (2020). Adenoid cystic carcinoma of the breast: a case report. Case Rep Oncol.

[5] Paintal A (2023). Pathology of head and neck neoplasms. UpToDate.

[6] Bleiweiss IJ (2023). Pathology of breast cancer. UpToDate.

[7] Zhang W, Fang Y, Zhang Z, Wang J (2021). Management of adenoid cystic carcinoma of the breast: a single-institution study. Front Oncol.

[8] Gomez-Seoane A, Davis A, Oyasiji T (2021). Treatment of adenoid cystic carcinoma of the breast: is postoperative radiation getting its due credit?. Breast.

[9] Boujelbene N, Khabir A, Boujelbene N, Jeanneret Sozzi W, Mirimanoff RO, Khanfir K (2012). Clinical review-breast adenoid cystic carcinoma. Breast.

[10] Arpino G, Clark GM, Mohsin S, Bardou VJ, Elledge RM (2002). Adenoid cystic carcinoma of the breast: molecular markers, treatment, and clinical outcome. Cancer.

[11] Lydiatt WM, Quivey JM (2023). Salivary gland tumors: treatment of locoregional disease. UpToDate.

